# Heparin induced thrombocytopenia-type 2

**DOI:** 10.4103/0973-6247.67028

**Published:** 2010-07

**Authors:** Tanveer A. Majeed

**Affiliations:** Department of Surgical Oncology, Mahavir Cancer Sansthan, Patna, Bihar, India

Sir,

Heparin-induced thrombocytopenia (Type II) is a rare and dangerous condition which occurs in 1% to 3% of patients receiving heparin. Thrombocytopenia occurs 5 to 10 days after heparin therapy decreasing the platelet counts by 30 to 50%.[1,2] Type II HIT primarily affects the venous system but arterial involvement is not uncommon. It leads to deep venous thrombosis, pulmonary embolism, shin necrosis, myocardial infarction, venous gangrene, and possibly death. Diagnosis is preceded by a high index of suspicion in a patient on heparin therapy with a progressive fall in platelet count. It is caused by formation of antibodies which activate the platelets following heparin administration. This leads to an interaction with platelet factor 4 (PF4) normally present on the endothelial cells and platelets, thus stimulating the formation of immunogenic P4 complexes which cause an immunogenic response. This complex leads to further activation of platelets-consequent formation of micropartides-thrombin generation which result in formation and embolization of thrombus.[1–3] These antibodies persist for weeks to months following heparin administration and hence re-exposure to heparin can induce a faster immunological reaction.[1] Clinical diagnosis of type II HIT can be made by C Serotonin release assay (SRA) and heparin-induced platelet activation assay (HIPAA). These tests are not freely available. Immunoglobulin’s---IgG, IgM, and IgA---antibodies are also detected. HIT specific antibodies could also be detected using ELISA.[1,4] These tests may be negative in some patients therefore a clinical suspicion is important to prevent thromboembolism.[1,2,3] Treatment options include inhibition of thrombin formation or direct thrombin inhibitors like bivaliurdin, lipirudin, and Argatroban. At present, only bivaliurdin is approved for use in India. It is for 5 days with ACT and PTT monitoring, and it is followed by overlap and maintenance therapy with Warfarin (Acetrome) 2 mg/day to maintain an INR of 2.5 to 3.0. Singer et al, evaluated 1500 patients who underwent CABG, and 0.75% patients developed HIT. The sequelae were as follows: 6 patients had to undergo amputations, 4 patients developed stroke, 2 patients developed myocardial infarction, 2 patients developed pulmonary emboli and 2 deaths, thus depicting the severity of this condition, if not diagnosed and treated early.[2] A high index of suspicion, timely diagnosis, medical and surgical management of thromboembolic phenomenon result in complete cure.
